# The *Rhododendron dauricum* L. Flavonoids Exert Vasodilation and Myocardial Preservation

**Published:** 2010

**Authors:** Bo-Nan Zhang, Yun-Long Hou, Bao-Jv Liu, Qing-Mei Liu, Guo-Fen Qiao

**Affiliations:** Department of Pharmacology, Harbin Medical University;194 Xuefu Road, Harbin 150081, China.

**Keywords:** *Rhododendron dauricum *flavonoids (RF), Vascular tone, Hypoxia, Myocardial preservation, Vasodilation

## Abstract

*Rhododendron dauricum *L*. *is an ancient Chinese traditional herb. The pharmacological effects of *R. dauricum *extract have been shown in chronic tracheitis. The aim of this study was to investigate the cardiovascular effects of *Rhododendron dauricum *L*. *flavonoids (RF) on rats and its mechanisms. This study was performed in isolated vascular rings and a rat model of myocardial infarction and isolated myocytes. RF (0.5 – 4 mg/mL) induced a concentration-dependent relaxant effect on the phenylephrine (10^-5^ M) and KCl (60 mM) contracted aortic rings, with or without intact endothelium. This effect was attenuated by pretreated with *L*-NAME (10^-5^ M) and K^+^ channel inhibitor 4 - AP (1 mM) and TEA (1 mM). The Ca^2+^-induced contraction and PE-induced contraction were obviously attenuated after pretreated with RF (2 mg/mL) for 30 min in Krebs solution, without Ca^2+^, containing 10^-4^ mol EGTA. KCl (60 mM) significantly increased the intracellular free Ca^2+^ concentration ([Ca^2+^]_i_) and RF inhibited the changes induced by KCl in single cardiac myocytes. RF obviously prolonged the survival time of hypoxia mice pretreated with isoprenaline and reduced the myocardial infarction size in rat coronary artery ligation. These findings suggest that RF induces concentration-dependent vasodilation and myocardial preservation.

## Introduction

Because of its low toxicity and good therapeutical performance, traditional Chinese medicine has attracted considerable attention for many fields. *Rhododendron dauricum *L*.*, referred to as “Man-shan-hong” in Chinese, belongs to the family of *Ericaceae*, the genera of *Rhododendron *L. *Rhododendron dauricum *L*. *is distributed in the northern part of China, and is a type of medicinal herb often used to cure chronic tracheitis. Chinese Pharmacopoeia (1977 edition) has listed *R. dauricum *as an official drug (1). Flavonoids are a kind of natural organic compounds with potential physiological activities and effective components of Chinese Herbs, which have attracted more and more research interest and exist in nature extensively. Numerous flavonoids have been reported in the leaves and twigs of *R. dauricum. *They are mainly composed of farrerol, flavonone, flavonol, flavone and flavonol glucoside (1). Pathological experiments have showed that farrerol moves phlegm and alleviates coughs, and farrerol has been synthesised chemically as an antibechic (2, 3). (Besides moving the phlegm and alleviating cough, other related investigations have shown that flavonoids could have a broad range of physiological activities such as anti- inflammatory, anti-bacterial and antioxidant activity for scavenging radicals). 

The mechanisms of *R. dauricum *flavonoids (RF) on respiratory system have been investigated, in which a decrease in [Ca^2+^]_i_ of bronchial smooth muscle seems to be involved. It is also well known that intracellular calcium-induced calcium release (CICR) is involved not only in bronchial and vascular smooth muscle, but also in myocardial cells, which could induce the smooth muscle and myocardial cell constriction. It has also been reported that [Ca^2+^]_i_ overload causes cell damage, as a result of cell undergoing hypoxic and ischemia. Should RF be capable of inhibiting influx of Ca^2+^ and intracellular calcium release in order to reduce [Ca^2+^]_i_, it would be able to avoid damage by calcium overload. 

Hence, the present study was designed to examine the effects of on the vascular tone and explore its cardiovascular protective effects, both *in vivo *and *in vitro*. 

## Experimental

*Extraction of RF*

*Rhododendron dauricum *(300 g) was extracted twice at 80-100°C, using 3000 mL water for 2 h each time. The extracts were then combined and filtered through filter paper. The filtrate was concentrated at 50-60°C by rotary evaporator to 1800 mL. This crude extract was then dissolved in three volumes 95% ethanol, under constant stirring. The ethanol suspension was laid overnight and then concentrated. Polyamide column chromatography (volume of 400 mL, and a speed of 400 mL/h) was used to enrich the flavonoid components. Non-flavonoid components were first eluted with 1200 mL distilled water and then 1500 mL 95 % ethanol was used to elute the flavonoid components, until reaching a negative reaction of hydrochloric acid-magnesium. The elution speed was about 400 mL/h. The flavonoids suspension was concentrated and then vacuum dried. The flavonoids powder was about 6 g and included hyperoside, quercetin, azaleatin and isorhamnetin. 

*Assay of flavonoids*

The experiments consisted of 3 parts.

*(І) Assay of hyperoside*

The condition of chromatography as below:

stationary phase: ODS volume (4.6 mm I.D. × 150 mm, 5 μm); mobile phase: methanol: 0.5% phosphoric acid (45: 55) aqueous solution (pH was adjusted with triethylamine to 3); speed was 0.8 mL/min; wave length of detection was 355 nm; and sample size was 10-20 μL. The content of hyperoside was 16.6%.

*(ІІ) Assay of azaleatin*

The condition of chromatography as below:

stationary phase: ODS volume (4.6 mm I.D. × 150 mm, 5 μm); mobile phase: methanol: water (60: 40); speed was 1 mL/min; wave length of detection was 296 nm; and sample size was 10-20 μL. The content of azaleatin was 1.1%.

*(ІІІ) Assay of quercetin*

The condition of chromatography as below:

stationary phase: ODS volume (4.6 mm I.D. × 150 mm, 5 μm); mobile phase: methanol: water: glacial acetic acid (50: 50: 2); speed was 1 mL/min; wave length of detection was 370 nm; and sample size was 10-20 μL. The content of quercetin was 3.8%.

*Preparation of thoracic arterial rings*

Male Wistar rats were purchased from Harbin Medical University animal center. The rats (each weighing 250 - 300 g) were sacrificed by decapitation. The thoracic aortas were rapidly and carefully dissected and placed into ice-cold and oxygen-saturated K-H buffer solution (pH=7.4) containing 118 mmol/l NaCl, 4.7 mmol/l KCl, 1.1 mmol/L MgSO_4_, 1.2 mmol/L KH_2_PO_4_, 1.5 mmol/L CaCl_2_, 25 mmol/L NaHCO_3_, and 10 mmol/L glucose. The thoracic aortas were cleaned of connective tissue, and cut into approximately 3-4 mm-wide strips. For endothelium-intact strips, extreme care was taken to avoid endothelium injury. For endothelium-denuded strips, the endothelium was removed by rubbing the vessel interior with wet filter paper. Each vascular strip was mounted in a 5-mL organ bath containing K-H buffer solution. One end of the aortic strip was attached to a metal hook and the other end was connected to an isometric force transducer (JH-2, metrical range: 0-10.0 g) in a bath containing K-H buffer solution, maintained at 37°C and bubbled with 95% O_2_ and 5% CO_2_. Rings were equilibrated for 45 min at 1.5 g resting tension, and then challenged with PE (1.0×10^-5^ M) until a maximal contractile response was obtained. The integrity of the endothelium was assessed in all preparations by determining the ability of carbachol (1.0×10^-5^ M), to induce more than 80% relaxation of rings. The endothelium was considered to be removed when there was less than 10% relaxation response to carbachol. The isometric tension was recorded with MedLab BL-420E^+^ recording system (Chengdu TME Technology Co., China).

*Effects of RF on vascular tone*

The experimental set up were consisted of 4 groups, with each group including six test animal.

*(I) Endothelium-dependent or independent effects of RF on isolated aortic rings*

When the tension was at resting state or reached a plateau induced by PE (1.0×10^-5^ M) or KCl (60 mM), RF (0.5, 1, 2, 3, 4 g/L) was cumulatively added into the organ bath at 4 min intervals. The rings with intact or denuded endothelium were always tested in parallel.

*(II) Effect of L-NAME (10* ^-5^ *M) pretreatment on RF induced vasorelaxation in rat aortic rings with endothelium*

Aortic rings were pretreated with NOS inhibitor *L*-NAME (10^-5^ M) 30 min before contraction with PE. Then RF (0.5, 1, 2, 3, 4 g/L ) was cumulatively added into the organ bath at 4 min intervals to examine a role of *L*-NAME. 

*(III) Effect of potassium channels blocking agents pretreatment on RF induced vasorelaxation in rat aortic rings without endothelium*

Aortic rings were pretreated respectively with potassium channels blocking agents, including 4-AP (1 mM), TEA (1 mM), Gli (10 μM) and BaCl_2_ (10 μM) 30 min before contraction with PE. Then RF (0.5, 1, 2, 3, 4 g/L) was cumulatively added into the organ bath at 4 min intervals to examine the role of potassium channel blocking agents.

*(IV) Effect of RF and KCl pretreatment in K-H buffer solution without Ca* ^2+^ *on Ca* ^2+^ *-induced contraction in isolated aortic rings*

Aortic rings were pretreated with RF (2 mg/mL) and KCl (60 mM) in K-H buffer solution without Ca^2+^ 30 min before contraction with Ca^2+^ (4). Then Ca^2+^ (0.5, 1, 2, 4, 8 mM) was cumulatively added into the organ bath at 4 min intervals to examine the role of RF on Ca^2+^- induced contraction.

*(V) Effect of RF pretreatment in K-H buffer solution without Ca* ^2+^ *on PE-induced contraction in isolated aortic rings*

Aortic rings were pretreated with RF (2 mg/mL) in K-H buffer solution without Ca^2+^ 30 min before contraction with PE. Then PE (1.0×10^-5^ M) was added into the organ bath at 4 min intervals to examine the role of RF on PE- induced contraction.

*Preparation of rat cardiomyocytes*

Myocytes were isolated according to the method established by Yang *et al. *(5). Briefly, each rat was killed, then the heart was quickly removed and cannulated on a Langendorff apparatus and retrogradely perfused through the aorta with the standard Tyrode’s solution (126 mM NaCl, 5.4 mN KCl, 10 mN HEPES 0.33 mN, NaH_2_PO_4_·2H_2_O 1.0 mN, MgCl_2_·6H_2_O, 1.8 mN CaCl_2_ and 10 mN glucose; pH=7.4) for 5 min, and calcium-free Tyrode’s solution until it stopped beating. The heart was then enzymatically digested with the calcium-free Tyrode’s solution, containing collagenase type II and BSA. The ventricular tissue was minced after being softened and placed in the KB medium (70 mM glutamic acid, 15 mN taurine, 30 mN KCl, 10mN KH_2_PO_4_, 10 mN HEPES, 5.4 mN MgCl_2_·6H_2_O, 10 mN glucose and 0.5 mN EGTA; pH=7.4). Single cells were obtained by gentle pipetting. It was stored at 4°C for 1–2 h, and then gassed with 95% oxygen and 5% carbon dioxide and warmed to 37 ± 0.5°C. Only rod-shaped myocytes with clear cross-striations were studied.

*[Ca* ^2+^ *]*_i_ *measurement*

After the isolation of single ventricular myocytes, they were adhered to the cover-slips of the chamber. Cells were then rinsed once with the normal Tyrode’s solution and subsequently incubated with a working solution containing Fluo-3/AM (20 mM) and Pluronic F-127 (0.03%) at 37°C for 45 min. After loading, the cells were washed once with the standard Tyrode’s solution to remove the extracellular Fluo- 3/AM. Fluorescent changes of the Fluo-3/AM-loaded cells were detected by a laser scanning confocal microscope at 488 nm for excitation from an argon ion laser and 530 nm for emission and inverted microscope with 20 × objective. Drugs were added between scans 3 and 4, and the images stored on disks. The fluorescent intensities, both before (F_0_) and after (F_i_) the drug administration, were recorded. The change in [Ca^2+^] _i_ was represented by the ratio of F _i_ /F_0_.

*Effect of RF on cardiomyocytes*

The experiments were carried out on 3 groups, each consisting of twenty test animal.

(І) Effect of KCl on [Ca^2+^]_i _

F _i_ was measured after adding KCl (60 mM) to the normal Tyrode’s solution containing 1.0 mmol/ L Ca^2+^.

(ІІ) Effect of RF on [Ca^2+^]_i_ elevation induced by KCl (60 mM) 

The preparation was pretreated with RF (1~2 mg/mL) for 10 min. F _i_ was then measured after adding 60mN KCl. 

(ІІІ) Effect of verapamil on [Ca2+]_i_ elevation induced by KCl (60 mM) 

The preparation was pretreated with calcium channel inhibitor verapamil (10 μM) for 10 min. F _i_ was then measured after adding 60 mM KCl.

*Effect of RF on hypoxia mice*

Fourty male mice (average weight of 20±2 g) were divided into 4 groups: 180 mg/kg RFH group, 90 mg/kg RFL group, control group (normal saline) and 10 mg/kg BN52021 group. Each group received intraperitoneal injection with isoprenaline (0.05 mg/10 g) 15 min after injecting drugs through vena caudalis and then each animal moved into a wide-mouthed bottle (180 mL) containing 10 g nitrica calx to examine the survival time, through the observation of asphyxia.

*Rat model of myocardial infarction*

Male Wistar rats weighing 230–250 g were randomly divided into five groups: control, ischemia (MI), ischemia-low RF (RFL, 7.5 mg/kg), ischemia-high RF (RFH, 30 mg/kg) and BN52021 (10 mg/kg), respectively. The rats were heparinized (300 U) and anesthetized with IP sodium pentobarbital (40 mg/kg), and were then ventilated using a small animal ventilator at a frequency of 70/min and a tidal volume of 3 mL. The chest was surgically opened and the body temperature was maintained at 37°C by placing the animal on a heating pad. The standard limb lead ECG, together with the arterial pressure, was continuously recorded on a recorder (Nihon Kohden RM 6200; Tokyo). A left thoracotomy was performed via the fourth rib intercostal space and a segment of saline-soaked 5-0 suture was looped around the left anterior descending (LAD) coronary artery, near its origin from the left coronary artery and then the chest was closed quickly.

*Measurements of infarction size*

The heart was removed from the animal after 60 min of infarction, and then the ventricular tissue was dissected and kept at −4°C overnight. The frozen ventricles were sliced into 2 mm thick sections, and then incubated in 1% triphenyltetrazolium chloride at 37°C in 0.2 M Tris buffer (pH=7.4) for 30 min. While normal myocardium was stained brick red, the infarcted areas remained unstained. The size of the infarcted area was estimated by its volume and weight, as a percentage

e of the left ventricle (6). 

*Statistical analysis*

Data were presented as mean ± SD. Statistical comparison was performed by the Student’s t - test and with an analysis of variance (ANOVA), with a value of p < 0.05 considered as significant. 

## Results

*Effects of RF on isolated aortic rings induced by PE and KCl*

**Figure 1:**
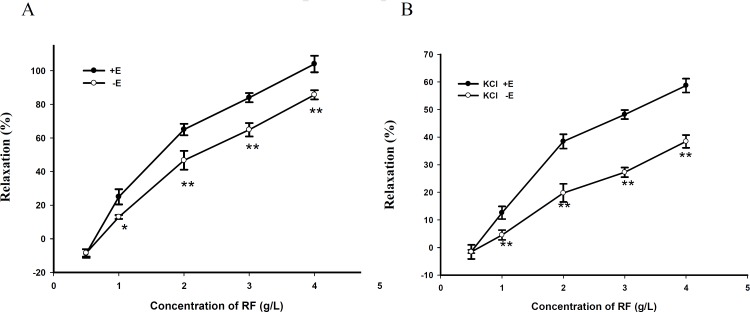
(A) Dose - dependent relaxant effects of RF contracted by PE on rat aortic rings with endothelium (+ E) or without endothelium (- E). Data are expressed as mean ± SD, (n=6); * p < 0.05 vs. + E, ** p < 0.01 vs. + E(B). Dose - dependent relaxant effects of RF contracted by KCl on rat aortic rings with endothelium (+ E) or without endothelium (- E). Data are expressed as mean ± SD, (n=6); (** p < 0.01 vs. + E).

RF relaxed PE (10^-5^ M)-precontracted aortic rings in a dose-dependent manner (Figure 1). 

Compared with endothelium - denuded rings, the maximal relaxant effect of RF on rings with endothelium was increased by about 20 ± 1.8% and RF also relaxed KCl (60 mM)-precontracted aortic rings in a dose-dependent manner. Compared with endothelium-denuded rings, the maximal relaxant effect of RF on rings with endothelium was increased by about 22 ± 3/AM. Fluorescent changes of the Fluo-3/AM-loaded cells were detected by a laser scanning confocal microscope at 488 nm for excitation from an argon ion laser and 530 nm for emission and inverted microscope with 20 × objective. Drugs were added between scans 3 and 4, and the images stored on disks. The fluorescent intensities, both before (F_0_) and after (F_i_) the drug administration, were recorded. The change in [Ca^2+^] _i _was represented by the ratio of F _i_ /F_0_.

**Figure 2:**
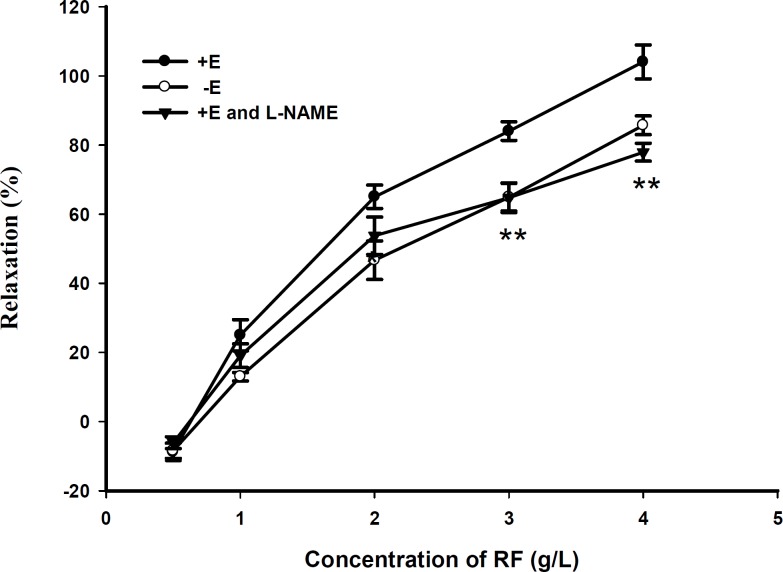
Effect of *L*-NAME (10^-5^ M) pretreatment on RF induced vasorelaxation in rat aortic rings with (+ E). Data are expressed as mean ± SD, (n=6); (** p < 0.01 vs. + E).

*Effect of RF on cardiomyocytes*

The experiments were carried out on 3 groups, each consisting of twenty test animal.

(І) Effect of KCl on [Ca^2+^]_i_

F _i_ was measured after adding KCl (60 mM) to the normal Tyrode’s solution containing 1.0 mmol/ L Ca^2+^.

(ІІ) Effect of RF on [Ca^2+^]_i_ elevation induced by KCl (60 mM) 

The preparation was pretreated with RF (1~2 mg/mL) for 10 min. F _i_ was then measured after adding 60mN KCl. 

(ІІІ) Effect of verapamil on [Ca^2+^]_i_ elevation induced by KCl (60 mM) 

The preparation was pretreated with calcium channel inhibitor verapamil (10 μM) for 10 min. F _i _was then measured after adding 60 mM KCl.

*Effect of RF on hypoxia mice*

Fourty male mice (average weight of 20±2 g) were divided into 4 groups: 180 mg/kg RFH group, 90 mg/kg RFL group, control group (normal saline) and 10 mg/kg BN52021 group. Each group received intraperitoneal injection with isoprenaline (0.05 mg/10 g) 15 min after injecting drugs through vena caudalis and then each animal moved into a wide-mouthed bottle (180 mL) containing 10 g nitrica calx to examine the survival time, through the observation of asphyxia.

*Rat model of myocardial infarction*

Male Wistar rats weighing 230–250 g were randomly divided into five groups: control, ischemia (MI), ischemia-low RF (RFL, 7.5 mg/kg), ischemia-high RF (RFH, 30 mg/kg) and BN52021 (10 mg/kg), respectively. The rats were heparinized (300 U) and anesthetized with IP sodium pentobarbital (40 mg/kg), and were then ventilated using a small animal ventilator at a frequency of 70/min and a tidal volume of 3 mL. The chest was surgically opened and the body temperature was maintained at 37°C by placing the animal on a heating pad. The standard limb lead ECG, together with the arterial pressure, was continuously recorded on a recorder (Nihon Kohden RM 6200; Tokyo). A left thoracotomy was performed via the fourth rib intercostal space and a segment of saline-soaked 5-0 suture was looped around the left anterior descending (LAD) coronary artery, near its origin from the left coronary artery and then the chest was closed quickly.

*Measurements of infarction size*

The heart was removed from the animal after 60 min of infarction, and then the ventricular tissue was dissected and kept at −4°C overnight. The frozen ventricles were sliced into 2 mm thick sections, and then incubated in 1% triphenyltetrazolium chloride at 37°C in 0.2 M Tris buffer (pH=7.4) for 30 min. While normal myocardium was stained brick red, the infarcted areas remained unstained. The size of the infarcted area was estimated by its volume and weight, as a percentage of the left ventricle (6). 

*Statistical analysis*

Data were presented as mean ± SD. Statistical comparison was performed by the Student’s t - test and with an analysis of variance (ANOVA), with a value of p < 0.05 considered as significant. 

*Effects of RF on isolated aortic rings induced by PE and KCl*

**Figure 3:**
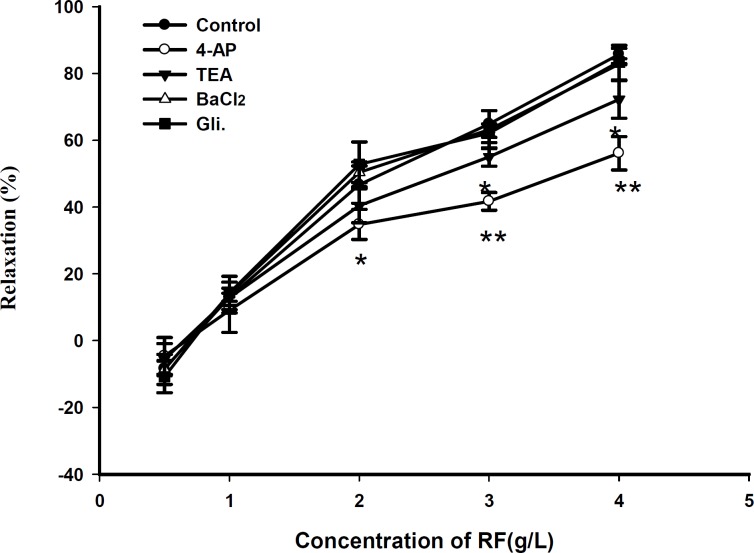
Effect of 4-AP (1 mM), TEA (1 mM), glibenclamide (10 μM), BaCl_2_ (10 μM) on RF - induced relaxation in endothelium - denuded aorta ring. Data are expressed as mean ± SD, (n=6); (* p < 0.05 vs. control, ** p < 0.01 vs. control

RF relaxed PE (10^-5^ M)-precontracted aortic rings in a dose-dependent manner (Figure 1). Compared with endothelium - denuded rings, the maximal relaxant effect of RF on rings with endothelium was increased by about 20 ± 1.8% and RF also relaxed KCl (60 mM)-precontracted aortic rings in a dose-dependent manner. Compared with endothelium-denuded rings, the maximal relaxant effect of RF on rings with endothelium was increased by about 22 ± channels (KATP) inhibitor glybenclamide (Gli) and the inward rectifier K^+^ channels inhibitor BaCl_2_ did not play a role on the relaxant effect of RF, compared with the control group (Figure 3).

*Effect of RF pretreatment in Krebs solution without Ca* ^2+^ *on Ca* ^2+^ *-induced contraction in isolated aortic rings*

**Figure 4:**
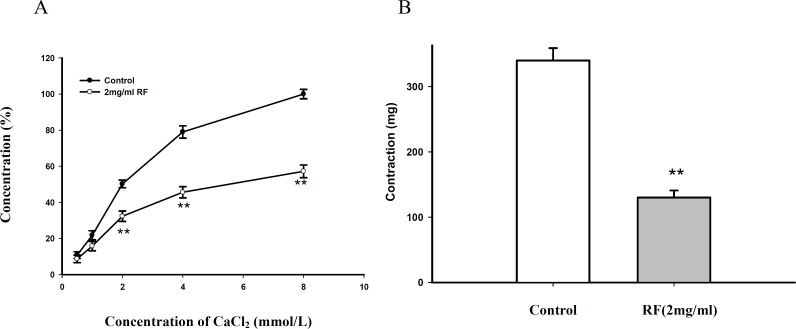
(A) Effect of RF on CaCl_2_ induced contraction in K - H without Ca^2+^. (B) Effect of RF on PE induced contraction in K - H without Ca^2+^. Data are expressed as mean ± SD, (n=6); (** p < 0.01 vs. control).

The Ca^2+^- induced contraction and PE induced contraction were obviously attenuated after pretreated with RF (2 mg/mL) for 30 min in Krebs solution without Ca^2+^ containing 10-4 mol EGTA, compared with the control group (Figure 4).

*Effect of RF on cardiomyocytes* *[Ca* ^2+^ *]*_i_

**Figure 5:**
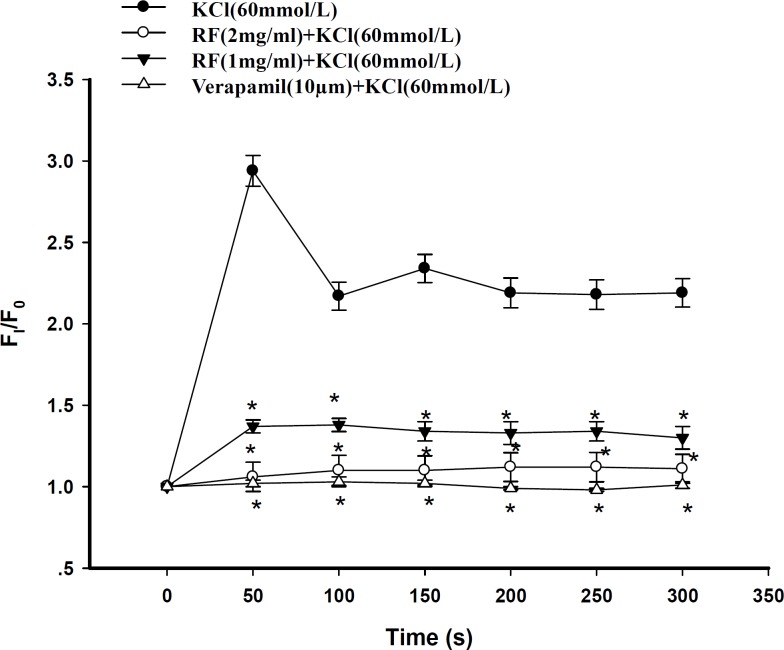
Dose-effect responses of verapamil and RF on increasing [Ca^2+^]_i_ induced by KCl. Verapamil 10 μm, RF 1 mg/mL and RF 2 mg/mL inhibited [Ca^2+^]_i _evoked by KCl, respectively. Verapamil or RF was applied 10 min before KCl (* p *< *0.05, versus KCl group, n=20 for each group).

**Figure 6:**
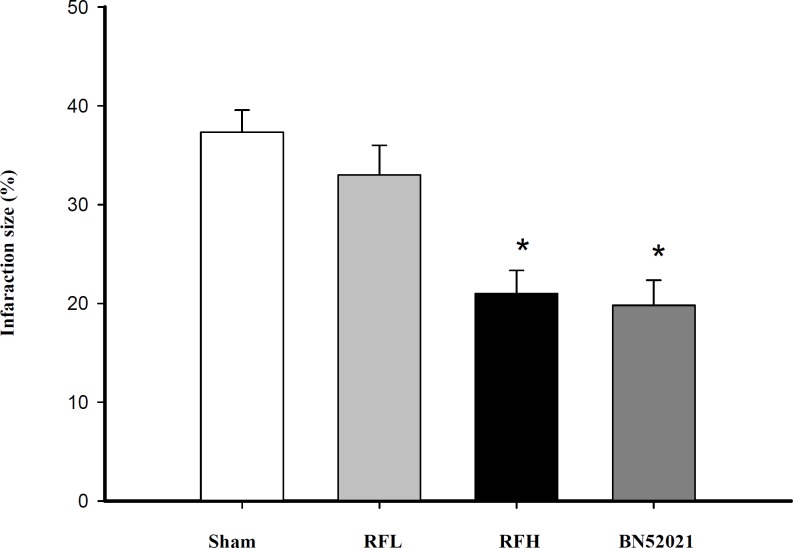
Effects of RF on infarcted size. Data are presented as mean of the infarcted size from the Sham, RFL (7.5 mg/kg RF, RFH (30 mg/kg RF), and 10 mg/kg BN52021 groups (* p *< *0.05 vs. sham, 10 rats in each group).

Prior to application of KCl, cardiac myocytes were incubated with either 10 μM verapamil or different doses of RF. Both verapamil and RF inhibited the increase in [Ca^2+^]_i_ induced by KCl. At the time point of 50s, KCl (60 mM) evoked a F _i _/F_0_ increase of 2.94 ± 0.11 fold, [Ca^2+^]_i_ (Figure 5). In contrast, in the group where cardiac myocytes were pre-incubated with RF, KCl had modest effects on the increase in [Ca^2+^]_i_. However, the inhibitory effect of RF on the increase in [Ca^2+^]_i_ induced by KCl was in a dose-dependent manner. As shown in Figure 6, the value of F _i_ /F_0_ in the presence of 1 mg/mL RF + KCl was 1.37 ± 0.04. In contrast, the value of F _i_ /F_0_ in the presence of 2 mg/mL RF plus KCl was 1.06 ± 0.09 ( p < 0.05, *n *= 20) (Figure 5).

*Effect of RF on survival time of hypoxia mice*

Compared with the control group, the mice survival time of 180mg/kg RFH group and the positive control group were prolonged by 4 min and 6 min (p < 0.05), respectively. This finding suggested that RF could obviously prolong the survival time in hypoxia mice (Table 1).

**Table 1:** Effect of RF on survival time of hypoxic mice (* p < 0.05, versus the control group, n=10 for each group).

**Group**	**Dose**	**Weight (g)**	**Time (min)**
Control	0.2 mL	20.15 ± 1.09	14 ± 5
RFH	180 mg/kg	19.45 ± 0.75	18 ± 3*
RFL	90 mg/kg	21.14 ± 1.47	15 ± 3
BN52021	10 mg/kg	20.20 ± 0.87	20 ± 4*

*Effect of RF on cardial infarction size during ischemia*

The results, as shown in Figure 6, indicated that the administration of RFL had a modest effect on the size of infarcted area of rat heart (reduced by 4.32 %, p > 0.05). However, the size of infarcted area was reduced by 16.14% at a higher concentration of RF (30 mg/kg). As a channels (KATP) inhibitor glybenclamide (Gli) and the inward rectifier K^+^ channels inhibitor BaCl_2_ did not play a role on the relaxant effect of RF, compared with the control group (Figure 3).

*Effect of RF pretreatment in Krebs solution without Ca* ^2+^ *on Ca* ^2+^ *-induced contraction in isolated aortic rings*

The Ca^2+^- induced contraction and PE induced contraction were obviously attenuated after pretreated with RF (2 mg/mL) for 30 min in Krebs solution without Ca^2+^ containing 10^-4^ mol EGTA, compared with the control group (Figure 4).

*Effect of RF on cardiomyocytes* *[Ca* ^2+^ *]*_i_

Prior to application of KCl, cardiac myocytes were incubated with either 10 μM verapamil or different doses of RF. Both verapamil and RF inhibited the increase in [Ca^2+^]_i_ induced by KCl. At the time point of 50s, KCl (60 mM) evoked a F _i_ /F_0_ increase of 2.94 ± 0.11 fold, [Ca^2+^]_i_ (Figure 5). In contrast, in the group where cardiac myocytes were pre-incubated with RF, KCl had modest effects on the increase in [Ca^2+^]_i_. However, the inhibitory effect of RF on the increase in [Ca^2+^]_i_ induced by KCl was in a dose-dependent manner. As shown in Figure 6, the value of F _i_ /F_0_ in the presence of 1 mg/mL RF + KCl was 1.37 ± 0.04. In contrast, the value of F _i_ /F_0_ in the presence of 2 mg/mL RF plus KCl was 1.06 ± 0.09 ( p < 0.05, *n *= 20) (Figure 5).

*Effect of RF on survival time of hypoxia mice*

Compared with the control group, the mice survival time of 180mg/kg RFH group and the positive control group were prolonged by 4 min and 6 min (p < 0.05), respectively. This finding suggested that RF could obviously prolong the survival time in hypoxia mice (Table 1).

*Effect of RF on cardial infarction size during ischemia*

The results, as shown in Figure 6, indicated that the administration of RFL had a modest effect on the size of infarcted area of rat heart (reduced by 4.32 %, p > 0.05). However, the size of infarcted area was reduced by 16.14% at a higher concentration of RF (30 mg/kg). As a positive control, 10 mg/kg of BN52021 was applied, which reduced the size of infarcted area of heart by 17.52% (Figure 6). 

## Discussion

*Rhododendron dauricum *is a type of medicinal herb often used to cure chronic tracheitis. The related literatures have demonstrated that the flavonoids present in the leaves of *R. dauricum *(such as Farrerol) could, relieve coughs and move phlegm. These flavonoids are thought to reduce the [Ca^2+^]_i_ , which can induce relaxation of tracheal smooth muscle and decrease gland excretion. There are several novel fingings in this present study: (i) the RF exerted a vasorelaxant effect on the phenylephrine and KCl contracted aortic ring with or without intact endothelium. (ii) Several mechanisms were involved, such as inhibition of influx of extracellular Ca^2+^ and release of Ca^2+^ from sarcoplasmic reticulum, contributing to NO release from intact endothelium, activation of voltage dependent K^+^ channels (K_V_) and calcium dependent K^+^ channels (K_Ca_). (iii) In the rat myocardial cells, incubation with RF could dose-dependently decrease [Ca^2+^]_i_ elevation by KCl, as observed by laser scanning confocal microscopy. (iv) In the animal experiments, RF could significantly prolong the hypoxia endurance time in mice and protect cardiac myocytes in the coronary artery ligation experiment. Based on these findings, we concluded that flavonoids are the active components of *Rhododendron dauricum *which can exert vasodilatation and myocardial preservation. The potentials of RF on cardiovascular disorder are closely related to a decreased [Ca^2+^]_i_ content in vascular smooth and cardiac muscle cells.

First of all, NO is a potent vasodilator synthesized in the endothelium by the enzyme NO synthase, and causes vascular smooth muscle cell relaxation through the activation of soluble guanylate cyclase (7). The present study demonstrated that RF, dose-dependently, inhibited the contraction induced by PE and KCl in intact aorta isolated from rats. This vasorelaxant action was partially inhibited by pretreatment with *L*-NAME. Endothelium-dependent relaxation of RF seemed to be associated with NO signaling, via guanylate cyclase activation, since *L*-NAME could attenuate this response. The direct endothelium-dependent and independent vasorelaxation should be taken into account, based on this study. Secondly, RF dose-dependently relaxed endothelium-denuded rings contracted with PE and KCl. The cellular mechanism of contraction involved in the response to KCl and PE is different. KCl induces Ca^2+^ influx via voltage-dependent Ca^2+^ channels, which further activates Ca^2+^-induced Ca^2+^ release through ryanodine-receptor. PE increases intracellular Ca^2+^ concentration via two mechanisms: (i) it activates receptor-gated Ca^2+^ channels, and (ii) it mobilizes Ca^2+^ from intracellular stores via the inositol-1, 4, 5- trisphosphate (IP_3_) receptor or induces secondly myosin light chain phosphorylation via activating PKC (8, 9). This means that, the vasorelaxant action of RF seems to occur in both the receptor-dependent and voltage-dependent manners in thoracic aortas. This hypothesis was confirmed through further experiments, in which RF inhibited the dose-independent contraction by CaCl_2_ and dose-independently decreased PE-induced vasocontraction in Ca^2+^-free Krebs solution. Thirdly, many types of K^+^ channels have been identified in vascular smooth muscle cells (VSMC) (10), and moreover, activated K^+^ channels induces hyperpolarization in order to reduce the [Ca^2+^]_i_ content in smooth muscle cells, which can exert vasodilatation. The TEA, a calcium-activated K^+^ channel inhibitor, and 4-AP, a voltage-dependent K^+^ channel inhibitor, weakened the vasodilatory effect of RF. But glibenclamide, an ATP-sensitive K^+^ channel inhibitor, and BaCl_2_, an inward rectifier of K^+^ channel inhibitor, had no influence on the vasodilatory effect of RF. It is concluded that RF could activate the voltage-dependent K^+ ^channel and the calcium-activated K^+^ channel and encourage the K^+^ efflux to reduce the [Ca^2+^]_i_ content in smooth muscle cells.

In this study, isolated vascular rings experiments indicated that RF-induced vasodilative effects partially contributed to the inhibition of [Ca^2+^]_i_, and these facts prompted us to testify the RF influence on isolated myocytes. Through the use of laser scanning confocal microscope, we observed that RF could dose-dependently inhibit the increase in [Ca^2+^]_i_ induced by KCl, and the inhibitory effect did not change with 10 μM verapamil at the concentration of 2 mg/mL. This result confirmed that RF functioned to inhibit the [Ca^2+^]_i_ on both vascular smooth muscle cells and myocytes, which supported the additional study, proposing that RF could exert cardioprotector effects. It has been proven that reducing the [Ca^2+^]_i_ content protects myocytes during damage by hypoxia and ischemia (11, 12). The results of his study indicated that RF prolonged survival time of hypoxic mice pretreated with isoprenaline and reduced the myocardial infarction size in rat coronary artery ligation, demonstrating that RF has a potential as a cardioprotector.

In conclusion, it could be said that explored the cadiovascular potency of RF in isolated myocytes, and vascular tissue in this study. Based on these studies, it could be said that so far the pharmacological activities of *R. dauricum *exceed that of the traditional pharmacodynamic limitations. Hence, RF seem to have the potential for development into a potent drug for use in the cardiovascular system.
